# Causes of Professional Ignorance

**Published:** 1862-04

**Authors:** 


### Selections.

CAUSES OF PROFESSIONAL IGNORANCE.—The following from an editorial on the above subject in a recent number of the American Medical Times will apply quite as well to the dental profession as the medical. We thought at first to make only some extracts from it, but in looking over it there was nothing we could afford to drop, and so we give it entire, suggesting for it close consideration.—ED. REG.

Often in reported cases mention is made of an "intelligent practitioner" who was called in an emergency, or was in some other way brought in contact with the patient. This term, now so often used as expressive of the qualifications of a particular physician, has more significance attached to it than one would at first be prepared to admit. Not every physician who claims the title has the right to the appellation of an intelligent one. This humiliating statement, we are sorry to say, can be corroborated by instances in every particular medical community. The existence of this class can be accounted for in a very great degree by the defective preliminary education so commonly met with among our students of medicine, rendering them altogether unfit for the proper elucidation of those abstruse points with which the science of the healing art abounds. Besides this, we have another very fruitful source of incompetency and ignorance, in the want of sufficiently rigid examinations by many of our medical colleges. The ordeal of the "Green room" is too often passed by the unqualified student without difficulty, and the physician, thus prematurely made and titled by law, claims what he considers his rightful rank. To these points, however, the attention of the profession has been frequently enough called, and no one doubts the necessity of a thorough reform in the whole matter of medical education. So much for the foundation which the profession has to work upon.

We wish at this time, in connection with the subject under consideration, to consider a very fruitful source of ignorance in our profession, and one which is important as applying equally to the educated and the illiterate, and that has reference to the want of a habit of systematic reading. It is indeed surprising to find how few physicians are given to reading any thing that treats of medical subjects; even those who have every seeming facility for improvement, in the shape of good libraries, scarcely ever have a disposition to look further into a new book than the heads of its chapters. In reference to the amount of reading done in the medical world, we may, for the sake of convenience, divide the profession into three general classes, giving a type of each.

Firstly, then, we notice the physician who has cultivated habits of study, and who is every spare moment with his books. His practice may be large, and his professional engagements numerous, yet he always finds time to spend with his favorite authors, and read with interest and profit his periodicals. Such men study because they have a passion for it; because they know its necessity, and because they are sure of its rewards. How we may well blush to own that we have too few of such among us.

Again, there is a class by no means small who have a disposition to study, but who are always finding something else of pressing importance to engage their attention. This habit of indolence, easily got into, is hard to shake off, and the victim becomes a constant prey to a knowledge of his ignorance on the one hand, and a desire for reputation on the other. These are not, however, wholly lost to their sense of duty, but are only thoroughly aroused when an emergency occurs in the shape of an important case. The book is then taken from the shelf, and the desired chapter is found. The result of the necessarily hurried reading is to render "confusion worse confounded;" a consultation ensues, and the chance for a reputation may probably be lost to him for ever. Can such a practitioner be unacquainted with the reason of his ignorance?

We will allude to still another class, who ignore reading almost altogether, and make it a practice to rely upon their general knowledge. The principles of medicine which were taught them during the time they attended medical lectures, they are content to spend a lifetime in putting in practice. Examples of such are frequently met with who for more than a quarter of a century have, as far as the acquirement of the knowledge of the science of their profession is concerned, made not the slightest advance. Were we to recite the many facts which have come to our knowledge as proofs of the ignorance of such men, we would be scarcely believed; and yet very many of them enjoy large practices, and their patients believe that they are receiving the benefits which all the recent improvements made in science can give them. It is impossible to conceive how any thing short of the merest luck can in the great majority of instances shield such from disgrace, while in their important cases we are forced to admit that they succeed as did the man who, when asked upon a certain occasion how he sent a bullet through the centre of a target, replied, "By shutting both my eyes."

We can not lose sight of the fact that the habit of careful reading must be practiced to a greater extent among medical men than it seems to be. No profession should embody more learning than does that of medicine, and the physician who neglects to improve opportunities for acquiring necessary knowledge is in the highest degree culpable.

The question of time is an excuse, which is perhaps oftenest urged against the proper prosecution of study—and with some show of reason. The uncertain calling of a physician no doubt interferes in a very great measure with his plans for self-improvement; a creature of emergency, he can call no time truly his own; but we will venture to say that even among the busiest, spare moments can always be found. The very class of cases which are the most destructive of time, the tedious labors, afford not unfrequently golden opportunities for study to him who prepares himself for it by a book in his pocket. It is said with a good deal of truth that busiest men have generally the most time, and no one need search very long in any community to find a case in point.

In large cities there is comparatively less reading among medical men generally than in small towns, the reason for which can easily be given by reference to the fact that consultations can be called at the shortest notice in the former places. The want of self-reliance is felt much more in the cities of New York, Philadelphia, and Boston, on account of the easy access which all have afforded to men in every specialty, than in the backwoods of civilization, where the unassuming country doctor can consult with no one but through his books. Had the city physician half the energy which is possessed by his country cousin, he could, on account of greater facilities, lay claim with good grace to superior qualifications. Surprise has often been expressed at the intelligence of some country physicians, but the trouble is not taken to inquire into the reason for it.

The question naturally comes up here, what shall we read? The answer is, standard works and medical periodicals. We have previously taken occasion to allude to the importance of the latter publications to all engaged in active practice. Standard works appear only at certain periods, while the office of the medical journal is essentially to keep the professional public informed upon every advance in science that has been made in the interval. We have no hesitation in saying that very many lives have been saved by a timely knowledge of a new and valuable mode of treatment, and we imagine that no prejudice against medical journals can offer an excuse for a neglect of duty to the patient.

In conclusion, we must once more urge this necessity upon all those who are not addicted to a habit of study—to set about earnestly to cultivate it, as a duty to themselves, their patients, and their profession. Scientific knowledge is within the reach of the humblest aspirant to medical honors, if he only have energy sufficient to put forth the requisite amount of untiring labor to obtain it. When every facility is afforded for improvement, indolence is the only excuse for ignorance. Let all those who are complaining of want of time, set about seriously to consider how very many opportunities they have allowed to slip by unnoticed. To those who are repentant of past indolence and are acting in accordance with their sense of duty, we would promise a rich reward; the habitual student, however, stands in need of no encouragement.

